# Novel regulatory therapies for prevention of Graft-versus-host disease

**DOI:** 10.1186/1741-7015-10-48

**Published:** 2012-05-15

**Authors:** Joseph Leventhal, Yiming Huang, Hong Xu, Idona Goode, Suzanne T Ildstad

**Affiliations:** 1 Comprehensive Transplant Center, Northwestern Memorial Hospital, Chicago, IL, USA; 2 Institute for Cellular Therapeutics, University of Louisville, Louisville, KY, USA

## Abstract

Graft-versus-host disease is one of the major transplant-related complications in allogeneic hematopoietic stem cell transplantation. Continued efforts have been made to prevent the occurrence of severe graft-versus-host disease by eliminating or suppressing donor-derived effector T cells. Conventional immunosuppression does not adequately prevent graft-versus-host disease, especially in mismatched transplants. Unfortunately, elimination of donor-derived T cells impairs stem cell engraftment, and delays immunologic reconstitution, rendering the recipient susceptible to post-transplant infections and disease relapse, with potentially lethal consequences. In this review, we discuss the role of dynamic immune regulation in controlling graft-versus-host disease, and how cell-based therapies are being developed using regulatory T cells and other tolerogenic cells for the prevention and treatment of graft-versus-host disease. In addition, advances in the design of cytoreductive conditioning regimens to selectively target graft-versus-host disease-inducing donor-derived T cells that have improved the safety of allogeneic stem cell transplantation are reviewed. Finally, we discuss advances in our understanding of the tolerogenic facilitating cell population, a phenotypically and functionally distinct population of bone marrow-derived cells which promote hematopoietic stem cell engraftment while reducing the risk of graft-versus-host disease.

## Review

### Regulatory T cells in graft-versus-host disease-prevention

Graft-versus-host disease (GVHD) remains a major obstacle for the clinical application of hematopoietic stem cell (HSC) transplantation [1]. GVHD is initiated by alloreactive donor T cells which recognize the host minor and major histocompatibility (MHC) antigens, proliferate, and damage target tissues. Donor T cells have been shown to enhance engraftment of HSC, reconstitute T cell immunity, and mediate a potent beneficial anti-tumor effect, known as graft-versus-leukemia (GVL) effect. Depletion of donor T cells impairs engraftment of HSC and abrogates the T cell-mediated GVL effect. In addition, administration of immunosuppressive drugs to prevent GVHD after HSC transplantation impairs T cell function and increases the risk of opportunistic infection and tumor relapse. Therefore, recent approaches have focused on tailored approaches to maintain the desirable effect of GVL yet avoid GVHD after HSC transplantation. Recent preclinical novel cell-based therapies have been developed to achieve these outcomes. They are currently being translated to the clinic.

The mechanisms of donor T cell (CD4^+ ^T cell and CD8^+ ^T cell)-mediated GVHD are multifactorial and include activation of macrophages and antigen-presenting cells (APC) by transplantation conditioning regimens to damage host tissue, releasing soluble cytokines such as TNF-α and IL-1; alloreactive T cell activation, proliferation and differentiation in response to host or donor APC; and alloreactive T cell infiltration and release of pro-inflammatory cytokines which leads to damage of the target tissue [2]. Over the past two decades, the importance of regulatory populations of lymphocytes in controlling immune responses has been increasingly appreciated. Although different cell subsets with regulatory activity have been described, including CD4^+^/CD25^+^/forkhead/winged helix transcription factor ^+ ^(FoxP3^+^), CD8^+^/CD28^-^, T/natural killer (NK) cells, and TCR^+^/CD4^-^/CD8^-^, most studies have concentrated on CD4^+^/CD25^+^/FoxP3^+ ^T cells [3]. Among the CD4^+ ^T cell population, CD4^+^/CD25^+^/FoxP3^+ ^regulatory T cells (T_reg_) have been demonstrated to suppress a variety of immune responses dependent on effector T cells.

CD4^+ ^T_reg _have been divided into two major groups: the naturally occurring T_reg _and inducible T_reg_. Both types of T_reg _have proven effective in preventing GVHD in murine models of GVHD [4,5] and, to a lesser extent, in human HSC transplantation [6-8]. Although studies have suggested that T_reg _downregulate both T helper 1- and T helper 2-mediated immune responses, mainly through IL-10 and transforming growth factor beta (TGF-β) production, direct cell-cell contact has also been postulated to be required as a mechanism of action. Natural T_reg _are generated in the thymus and are nonspecific in their suppressive capability [3,9]. Although natural T_reg _must encounter antigens to exert their suppressive effects, once activated they suppress in an antigen-nonspecific manner, presumably through the release of immunosuppressive cytokines such as IL-10 and TGF-β [10]. Because of their nonspecific mechanism of action, there is concern regarding their clinical relevance. Importantly, antigen-specific T_reg _are inducible and need to be activated through their TCR in order to mediate their suppressive activities. The expression of receptors of chemokines, such as C-C chemokine receptor type 5 (CCR5) and CXC chemokine receptor 3 (CXCR3), on antigen-specific T_reg _support a role for proper trafficking of T_reg _to target tissue in the prevention of acute GVHD in murine models [11,12]. A recent study showed that tolerant patients without GVHD after HSC transplantation expressed significantly higher levels of CCR5 and CXCR3 compared with patients with acute GVHD early after HSC transplantation [8], suggesting that homing of T_reg _to secondary lymphoid tissues and sites of inflammation may play an important role in the control of GVHD.

Several studies in experimental murine models have shown that T_reg _can suppress proliferative activity of conventional CD4^+ ^T cells and CD8^+ ^T cells to alloantigenic stimulation *in vitro *and induce transplantation tolerance and reduce acute GVHD occurrence *in vivo *[13-15]. Donor CD4^+^/CD25^+ ^T_reg _isolated from the spleen or bone marrow of C57BL/6 mice can suppress lethal GVHD induced by transplanted donor CD4^+^/CD25^- ^T effector cells after allogeneic T cell-depleted bone marrow transplantation (BMT) [14]. Importantly, the beneficial effect of adoptively transferred CD4^+^/CD25^+ ^T_reg _does not abrogate the beneficial effect of donor T cell-mediated GVL [13]. Ianni *et al. *recently evaluated the impact of early infusion of freshly isolated human donor CD4^+^/CD25^+ ^T_reg _followed by conventional T cells in human leukocyte antigen (HLA)-haploidentical HSC transplantation [16]. Adoptive transfer of human donor T_reg _prevented acute as well as chronic GVHD in the absence of immunosuppressive drug therapy after transplantation. Moreover, T_reg _promoted immune reconstitution of CD4^+ ^T cells, CD8^+ ^T cells, B cells, and NK cells, and improved protective immunity against cytomegalovirus infection.

The major limitation for the clinical application of T_reg_-based therapy to prevent GVHD is obtaining sufficient numbers of antigen-specific T_reg _and maintaining their regulatory properties after infusion [17]. Studies showed that *ex vivo *expansion of mouse recipient-specific CD4^+^/CD25^+ ^T_reg _can be obtained using donor T_reg _stimulation with allogeneic recipient APC [18,19]. *Ex vivo *expansion of T_reg _resulted in specific tolerance for recipient-type alloantigens, but not for third-party antigens. Moreover, the T_reg _effectively controlled GVHD while favoring immune reconstitution and maintaining GVL in a murine model [18]. Another study showed that rapamycin-induced T_reg _from human CD4^+^/CD25^-^/CD45RA^+ ^cells exhibit a potent suppressive function *in vitro *compared with natural T_reg_, and suppress acute GVHD in a xenogeneic NOD/SCID mouse model [20]. Clinical trials of therapeutic cell transfer using T_reg _for the prevention and/or treatment of GVHD have recently been described. Ianni *et al. *reported that the adoptive transfer of freshly isolated, donor-derived natural T_reg _followed by conventional donor-derived T cells (Tcon) at the time of full-haplotype-mismatched adult HSC transplantation in patients being treated for hematologic malignancies prevented acute or chronic GVHD while favoring Tcon-mediated post-transplantation immune reconstitution [16]. Brunstein *et al. *detailed a 'first-in-human' phase 1 clinical trial of nonspecific (CD3/CD28 bead)-based *ex vivo *expanded umbilical cord blood-derived T_reg _in patients after umbilical cord blood transplantation and noted a reduced incidence of severe acute GVHD [21].

T_reg _as diagnostic and prognostic cellular biomarkers for acute GVHD have been investigated in mouse and rat models and human HSC transplantation [22-25]. Studies reported that the level of FoxP3 mRNA expression in peripheral blood mononuclear cells from patients with either acute GVHD or chronic GVHD was significantly decreased compared with patients without GVHD [25,26], suggesting that a regulatory mechanism is involved in the development of GVHD. The number of FoxP3^+ ^T_reg _in skin biopsies from GVHD patients was significantly higher in patients responding to GVHD treatment and with a lower grade of GVHD compared with those with more severe GVHD [23]. Taken together, these observations indicate that T_reg _can regulate immune responses and induce tolerance to alloantigen after HSC transplantation. These findings provide evidence for the use of T_reg _as a useful biologic to improve GVHD diagnosis and treatment.

### Cyclophosphamide and prevention of graft-versus-host disease

In GVHD, functional immune cells in the donor bone marrow recognize the recipient as foreign and mount an immunologic attack on the patient's organs and tissues, impairing their ability to function [1]. Strict HLA matching between donors and transplant patients has been proven to reduce the incidence and severity of GVHD [27]. However, approximately 50% of patients still develop GVHD even if they undergo allogeneic HSC transplantation with HLA-matched donors [28]. The challenge facing HSC transplantation is to achieve better allogeneic engraftment without graft rejection and GVHD. The common reagents of GVHD prophylaxis comprise cyclosporine, or tacrolimus, methotrexate, mycophenolic acid, or sirolimus [29-31]. However, cyclophosphamide has been found more recently in animal and clinical studies to be a better and potent prophylactic agent of GVHD without obvious aplasia due to toxicity to donor stem cells [32-36]. When it is used in high dose after allogeneic BMT in mouse models, cyclophosphamide targets alloreactive proliferating T cells and enhances alloengraftment without causing GVHD [32-34]. Therefore, in order to gain the effect of cyclophosphamide in enhancing alloengraftment, it has to be used 48 hours after BMC infusion but not before [32]. High-dose cyclophosphamide given on day 2 after transplantation mitigated both the incidence and severity of acute GVHD in an MHC-mismatched donor-recipient combination [37]. The effect of post-transplantation cyclophosphamide in GVHD prevention has been demonstrated by inhibiting graft-versus-host reactions and selectively eliminating activated T cells in response to donor antigens in minor histocompatibility disparity [38] and MHC-mismatched [33] mouse models. Clinically, post-transplantation cyclophosphamide has reduced the incidence and severity of GVHD across MHC barriers after BMT [33-35,39,40]. High expression of aldehyde dehydrogenase in HSC allows for the prompt inactivation of cyclophosphamide and helps explains their relative resistance to the effects of this alkylating agent. In contrast, activated lymphocytes express low levels of aldehyde dehydrogenase and are particularly susceptible to cyclophosphamide. Importantly, these preclinical studies demonstrated the specific safety profile of cyclophosphamide in stem cell-sparing activity, thus provided the preclinical rationale to proceed with a clinical trial of partially HLA-mismatched or HLA-haploidentical BMT with high-dose post-transplantation cyclophosphamide for patients with poor prognosis hematologic malignancies.

The major clinical rationale for HLA-haploidentical BMT is to extend the potential benefits of HSC transplantation and the graft-versus-tumor effect to patients who lack an HLA-matched donor. The major challenge is to reduce the incidence of fatal graft rejection, severe GVHD and treatment-related mortality while promoting engraftment. Luznik *et al. *recently reported the outcomes of 68 patients with poor-risk hematologic malignancies who were conditioned with fludarabine, cyclophosphamide and 2 Gy total body irradiation prior to receiving T cell-replete bone marrow from HLA-haploidentical, first-degree relatives [36]. Donors and recipients were mismatched at a median of four out of five HLA alleles, indicating substantial donor-recipient histoincompatibility. GVHD prophylaxis comprised intravenous cyclophosphamide 50 mg/kg on day 3 (n = 28) or on days 3 and 4 (n = 40) after transplantation, followed by tacrolimus and, mycophenolic acid. These two drugs were included to provide extra protection against graft failure and GVHD. They were started after the completion of cyclophosphamide in light of preclinical data indicating that calcineurin inhibitors block cyclophosphamide-induced transplantation tolerance by blocking the activation and proliferation of alloreactive T cells [41]. Recovery of neutrophils and platelets occurred at a median of 15 and 24 days, respectively, after transplantation. Graft failure occurred in nine patients (13%). Acute grades II to IV occurred in 34% and III to IV GVHD in 6% of patients, and chronic GVHD developed in 15% of patients. These rates of GVHD development are comparable to or below the incidences of acute and chronic GVHD after HLA-matched HSC transplantation without post-transplantation cyclophosphamide [42]. One year after transplantation, the cumulative incidence of relapse mortality was 51% and non-relapse mortality was 15%, and overall survival and event-free survival at 2 years after transplantation were 36% and 26%, respectively. Only six patients died, four of infection and two of GVHD. These data support the safety and potential efficacy of post-transplantation cyclophosphamide in preventing GVHD and fatal graft rejection after HLA-haploidentical HSC transplantation.

Based upon the results of Luznik and Fuchs [35,36,39], we have included the use of post-transplant cyclophosphamide (50 mg/kg day, 3 days after BMT) as part of a non-myeloablative conditioning regimen being used in an ongoing phase 2 trial of tolerance induction in recipients of mismatched kidney and stem cell transplants. Durable donor chimerism has been achieved in five of the first eight participants and no patient has developed GVHD [43].

### The facilitating cell: a key regulator of stem cell engraftment

T cell depletion (TCD) of allogeneic bone marrow is extremely effective at reducing the risk of GVHD, but greatly increases the risk of engraftment failure. Three possible reasons for TCD-associated-engraftment failure have been hypothesized. First, HSC can engraft across MHC-matched but minor disparate barriers, but fail to engraft in allogeneic recipients because they are harmed or removed during TCD. Second, T cells act as essential partner cells to promote allogeneic HSC engraftment. Third, an additional bone marrow population that shares some T cell markers protects the HSC from rejection or 'facilitates' HSC engraftment, but is inadvertently removed as an 'innocent bystander' during TCD. Investigations in different laboratories have provided strong evidence in support of the latter hypothesis. Initial studies in mice using different cell subsets sorted from donor bone marrow identified a distinct population of CD8^+^/TCR^- ^facilitating cells (FC) capable of promoting allogeneic HSC engraftment and establishment of mixed chimerism [44]. The addition of 30,000 MHC-matched purified FC to as few as 10,000 donor HSC into ablated MHC-disparate B10 recipients resulted in stable allogeneic engraftment across both class I and class II barriers, without causing GVHD [44]. Transplantation of FC alone failed to rescue recipients, indicating that FC are not an HSC. Importantly, FC + HSC reconstituted animals exhibit donor-specific tolerance for skin, cardiac and islet grafts.

**Figure 1:**
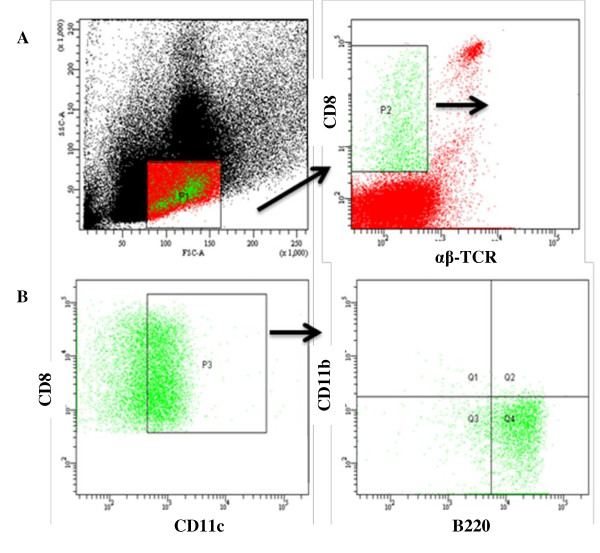
**CD8^+^/TCR^- ^facilitating cells**. Flow cytometric analysis of bone marrow FC stained with CD8α phycoerythrin, αβ-TCR fluorescein isothiocyanate, and γδ-TCR fluorescein isothiocyanate mAb. **(A) **FC comprised approximately 0.4% (range, 0.04% to 0.62%) of the total bone marrow and less than 1.6% of cells in the lymphoid gate. **(B) **The majority of cells in the FC are CD11c^+^/B220^+^/CD11b^- ^p-preDC. FC: facilitating cells; p-preDC: plasmacytoid precursor dendritic cells; TCR: T cell receptor.

In light of the fact that FC function is distinct from that demonstrated by bone-marrow-derived mature T cells, much research has gone into identifying the resident cell subpopulations that constitute this rare facilitating population. Previous studies have shown that the CD8^+^/TCR^- ^FC population shares phenotypic characteristics with CD8α plasmacytoid precursor dendritic cells (p-preDC) and that total FC are heterogeneous in morphology [45]. A comprehensive assessment of FC surface markers using flow cytometric analysis of sorted FC populations by Fugier-Vivier *et al. *[46] revealed a broad heterogeneity in subpopulations with facilitating potential. The majority of CD8^+^/TCR^- ^FC were positive for CD11c^+ ^(65% to 70%) and B220^+ ^(75% to 88%) expression. While only 15% of the B220^+ ^population was composed of B cells, 55% co-expressed CD11c (CD11c^+^/B220^+^), suggestive of a dendritic cell component. Further analysis into this CD11c^+ ^dendritic subset revealed that a p-preDC phenotype (CD11^dim^/B220^+^/CD11b^-^) is the predominant cell type (93% to 95%) within this CD11c^+ ^subset (Figure 1). The functionality of this p-preDC-like subset of FC has been assessed by transplanting these cells with HSC into ablated murine allogeneic recipients. The co-transplantation of p-preDC rescues allogeneic recipients from radiation aplasia, significantly enhancing alloengraftment and survival compared with ablated recipients that received HSC alone. However, facilitation by this p-preDC component alone was inferior to that observed when the entire FC population was utilized. This observation suggests that the p-pre DC subset is necessary, but not sufficient, for HSC facilitation and cannot fully replace the total FC population. Other elegant studies by Taylor *et al. *indicate that CD3e expression by FC and the association of CD3e with TCRβ chain and a novel 33 kDa protein termed FCp33 is critical for the HSC engraftment enhancing properties of FC [47].

Recent studies have focused on exploring FC and seeking to understand their role in generating tolerance. One such study completed on mice consisted of three groups of purified bone marrow inocula, which contained different subsets of CD8^+ ^cells that were administered to semi-allogeneic recipient mice [48]. The first group was αβ-TCR^+ ^splenic T cells, the second group was the CD8^+^/TCR^+ ^bone-marrow-derived T cells, and the third group was the bone-marrow-derived CD8^+^/TCR^- ^FC population. The purpose behind this experimental setup was to determine which group of cells would induce GVHD in the recipient mice. There were several important findings that resulted from this study [48]. The first was disproving the common belief that administering donor CD8^+ ^T cells would enhance engraftment and prevent the development of GVHD. This idea is supported by the fact that mice in the first and second groups of the experiment developed lethal GVHD, while mice in the group that received FC failed to develop clinical symptoms or histological evidence of GVHD [48]. Additionally, the researchers reported failure of FC to induce GVHD in mice when the number of FC transplanted was increased from 50,000 to 400,000, confirming that FC lack GVHD effector activity. Researchers also explored the mechanism behind the lack of GVHD development with transplantation of FC versus transplantation of the CD8^+^/TCR^+ ^bone marrow cells [48]. The results from such studies found an increase in the expression of T-cell-mediated immunosuppression factors. In particular, mice that received HSC with FC showed a significant increase in the transcription of TGF-β, a cytokine known to be a key player in the induction and development of T_reg_. There was also a considerable increase in the expression of the cyte-associated antigen 4, glucocorticoid-induced TNF receptor and FoxP3 genes, which are associated with T_reg _and suppressor T cell function. It was concluded that FC induce the formation of immunoregulatory T cells *in vivo*, since the FC is not a T_reg _and does not express the genes linked to T_reg _[48].

The ability of FC to induce the formation of CD4^+^/CD25^+ ^T_reg _from naive CD4^+^/CD25^- ^T cells, as shown in one recent study, is another proposed mechanism of how FC promote tolerance [49]. CD4^+^/CD25^+ ^T_reg _cells have been found to promote bone marrow engraftment and chimerism when transplanted into irradiated recipients [49]. Additionally, CD4^+^/CD25^+ ^cells are thought to prevent the development of GVHD after allogeneic transplantation. Both of these observations are similar to those observed with BMT containing FC. This has lead researchers to consider an association between FC and CD4^+^/CD25^+ ^T_reg _in inducing tolerance and preventing GVHD [49].

**Figure 2:**
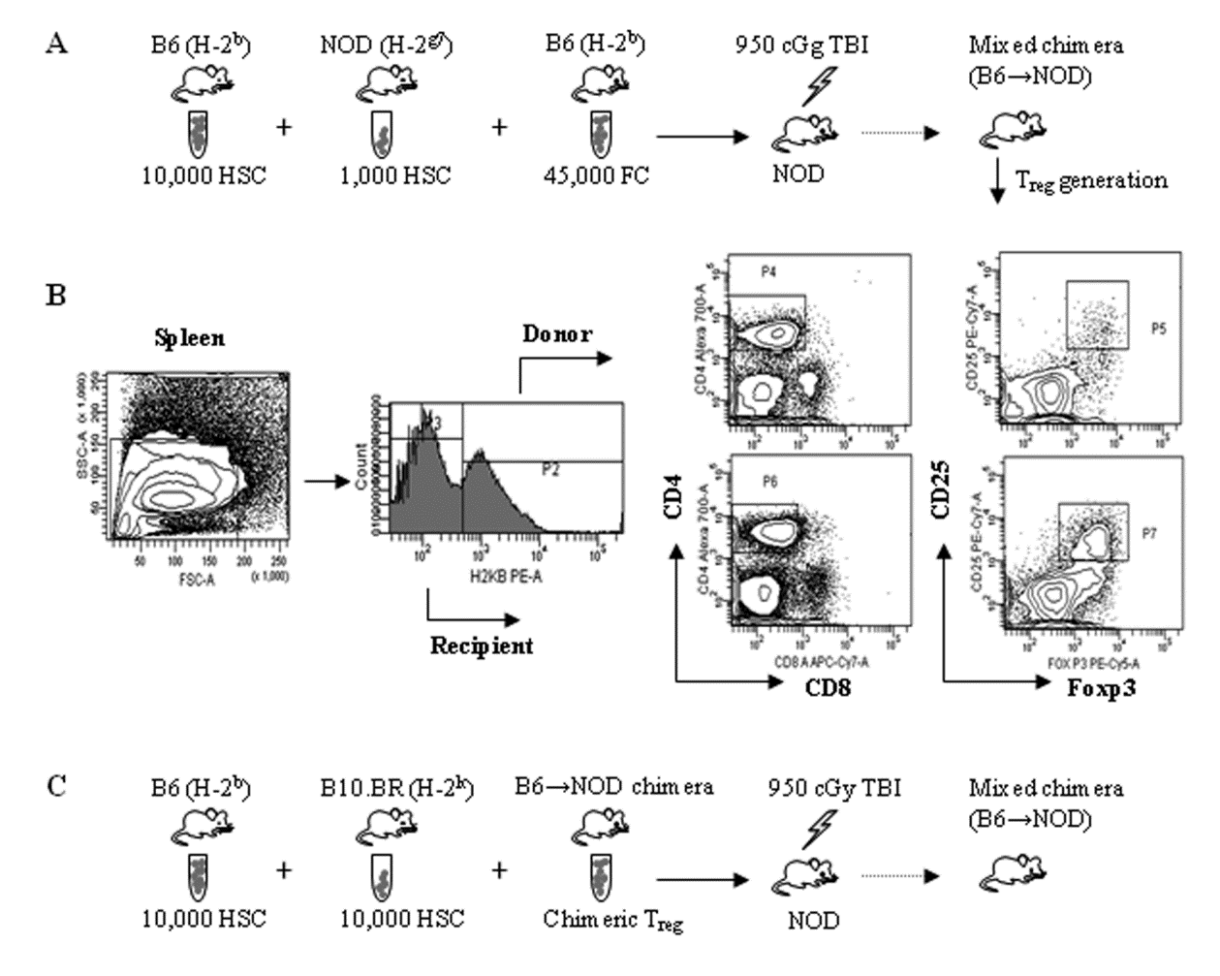
**Facilitating cells induce regulatory T cell generation**. **(A) **In B6 → NOD allogeneic model, NOD recipients were conditioned with 950 cGy of total body irradiation and transplanted with purified B6 HSC and NOD HSC with B6 FC via lateral tail vein injection. **(B) **Five weeks after transplantation, donor or recipient CD8^-^/CD4^+^/CD25^+^/FoxP3^+ ^T_reg _in chimeric spleen were analyzed. (This research was originally published in: Huang Y, Bozulic LD, Miller T, Xu H, Hussain L-R, Ildstad ST. CD8α^+ ^plasmacytoid precursor DC induce antigen-specific regulatory T cells that enhance HSC engraftment *in vivo*. Blood 2011, 17(8):2494-2505 with kind permission from The American Society of Hematology. Figure 2A has been modified.) **(C) **Chimeric T_reg _were transplanted with donor-specific B6 HSC and third-party B10.BR HSC into conditional NOD recipients. Chimeric T_reg _enhance HSC engraftment in an antigen-specific fashion. FC: facilitating cells; HSC: hematopoietic stem cell; T_reg_: regulatory T cell.

It was believed that p-preDC were the primary activators of naive CD4^+^/CD25^- ^T cells; however, Taylor *et al. *have shown that FC possess this function as well [49]. The Taylor study involved mice receiving an allogeneic HSC plus FC transplant and measuring the induction of FoxP3^+^/CD4^+^/CD25^+ ^by FC. Three major observations were made by this group that expanded the scientific knowledge of FC. The first was that donor FC were observed in the spleen shortly after transplantation and the induction of donor FoxP3^+^/CD4^+^/CD25^+ ^T_reg _also occurred in the spleen. Since p-preDC activate T_reg _in the spleen, the presence of FC in the spleen prior to activation of T_reg _supports the notion that FC activate T_reg_. The second was the observation that FC could induce the formation of FoxP3^+^/CD4^+^/CD25^+ ^T_reg _from CD4^+^/CD25^- ^T cells in co-culture. The researchers proposed a mechanism of activation of FC similar to that of p-preDC, to generate FoxP3^+^/CD4^+^/CD25^+ ^T cells. However, higher levels of IL4 gene expression were observed with FC-generated CD4^+^/CD25^+ ^T cells in comparison with p-preDC-generated T cells [49]. The last significant observation was that FC required direct cell-to-cell contact to induce the formation of FoxP3^+^/CD4^+^/CD25^+ ^T_reg_. Fewer FoxP3^+^/CD4^+^/CD25^+ ^cells were produced in the absence of physical contact than when direct contact occurred between the FC and the CD4^+^/CD25^- ^T cells [49]. Recent studies have suggested that the differentiation of CD4^+^/CD25^- ^T cells into CD4^+^/CD25^+ ^T cells requires stimulation from various sources [49]. The B7 receptor is one important aspect of this activation process since studies have demonstrated a lower number of CD4^+^/CD25^+ ^T cells in mice with a B7 deficiency [49]. CD86 is thought be another important player in assisting FC to produce FoxP3^+^/CD4^+^/CD25^+ ^T_reg _because its presence is required for differentiation [49]. More recently, FC have been shown to induce antigen-specific T_reg _*in vivo *(Figure 2) [50]. Transplantation of FC plus HSC into conditioned allogeneic mouse recipients followed by harvest of CD4^+^/CD25^+ ^T_reg _from the spleen of chimeras (chimeric T_reg_), and secondary transplantation of the T_reg _with donor HSC plus third-party HSC resulted in engraftment of only the donor HSC, demonstrating antigen-specificity.

In recent years there have been significant advances in understanding the mechanism of FC function, which have provided additional support for the promising potential for FC to be used in the clinic to generate tolerance after HSC transplantation. Data collected from recent studies have helped to distinguish FC from other hematopoietic cells such as p-preDC. However, there are questions that remain to be answered regarding the role of FC in inducing tolerance, preventing GVHD and promoting chimerism following BMT. A phase 2 clinical study approved by the Food and Drug Administration is currently underway to test the use of FC-based HSC transplantation in recipients of living donor renal allografts. Preliminary findings have demonstrated that high levels of donor macrochimerism without GVHD can be established in mismatched recipients conditioned with fludarabine, cyclophosphamide and 200 cGy total body irradiation [51].

## Conclusion

The use of cell-based therapies in the prevention and treatment of GVHD is now being applied in a number of clinical protocols, with promising results. Cell-based therapies have the potential to promote engraftment, preserve immunocompetence and prevent GVHD, thereby avoiding the toxicity of broad acting nonspecific immunosuppressive agents.

## Abbreviations

APC: antigen-presenting cells; BMT: bone marrow transplantation; CCR5: C-C chemokine receptor; CXCR3: CXC chemokine receptor 3; DC: dendritic cells; FC: facilitating cells; FoxP3: forkhead/winged helix transcription factor; GVHD: graft-versus-host disease; GVL: graft-versus-leukemia; HLA: human leukocyte antigen; HSC: hematopoietic stem cell; IL-1: interleukin-1; MHC: major histocompatibility complex; NK: natural killer; p-preDC: plasmacytoid precursor dendritic cells; TCD: T cell depletion; Tcon: conventional donor-derived T cells; TCR: T cell receptor; TGF-β: transforming growth factor beta; T_reg_: regulatory T cell; TNF-α: tumor necrosis factors-α; UCB: umbilical cord blood.

## Competing interests

STI has significant equity interest in Regenerex, LLC, a start-up biotechnology company based on the facilitating cell technology. All other authors have no conflict of interest to declare.

## Authors' contributions

JL: overall preparation and editing of review; YH: studies on role of T_reg _cells in GVHD prevention; HX: studies on role of cyclophosphamide; IG: review of FC mechanism of action; STI: overall editing and preparation of review. All authors have read and approved the final manuscript.

## Pre-publication history

The pre-publication history for this paper can be accessed here:

http://www.biomedcentral.com/1741-7015/10/48/prepub
